# Exercise and Metformin Intervention Prevents Lipotoxicity-Induced Hepatocyte Apoptosis by Alleviating Oxidative and ER Stress and Activating the AMPK/Nrf2/HO-1 Signaling Pathway in db/db Mice

**DOI:** 10.1155/2022/2297268

**Published:** 2022-09-09

**Authors:** Yuan Zhang, Yuting Liu, Xiaowei Liu, Xinmeng Yuan, Mengqi Xiang, Jingjing Liu, Liumei Zhang, Shouqian Zhu, Jiao Lu, Qiang Tang, Sulin Cheng

**Affiliations:** ^1^School of Sports and Health, Nanjing Sport Institute, Nanjing 210014, China; ^2^Department of Physical Education, Shanghai Jiao Tong University, Shanghai 200240, China; ^3^Jiangsu Collaborative Innovation Center for Sport and Health Project, Nanjing 210014, China; ^4^The Exercise Translational Medicine Centre, and the Key Laboratory of Systems Biomedicine, Ministry of Education, Shanghai Center for Systems Biomedicine, Shanghai Jiao Tong University, 200240, Shanghai, China; ^5^Huishan District Rehabilitation Hospital, Wuxi 214100, China; ^6^Faculty of Sport and Health Sciences, University of Jyväskylä, Jyväskylä 40014, Finland

## Abstract

**Objective:**

Nonalcoholic fatty liver disease (NAFLD) and type 2 diabetes (T2DM) commonly coexist and act synergistically to drive adverse clinical outcomes. This study is aimed at investigating the effects of exercise intervention and oral hypoglycaemic drug of metformin (MET) alone or combined on hepatic lipid accumulation. To investigate if oxidative stress and endoplasmic reticulum stress (ERS) are involved in lipotoxicity-induced hepatocyte apoptosis in diabetic mice and whether exercise and/or MET alleviated oxidative stress or ERS-apoptosis by AMPK-Nrf2-HO-1 signaling pathway.

**Methods:**

Forty db/db mice with diabetes (random blood glucose ≥ 250 mg/dL) were randomly allocated into four groups: control (CON), exercise training alone (EX), metformin treatment alone (MET), and exercise combined with metformin (EM) groups. Hematoxylin-eosin and oil red O staining were carried out to observe hepatic lipid accumulation. Immunohistochemical and TUNEL methods were used to detect the protein expression of the binding immunoglobulin protein (BiP) and superoxide dismutase-1 (SOD1) and the apoptosis level of hepatocytes. ERS-related gene expression and the AMPK-Nrf2-HO-1 signaling pathway were tested by western blotting.

**Results:**

Our data showed that db/db mice exhibited increased liver lipid accumulation, which induced oxidative and ER stress of the PERK-eIF2*α*-ATF4 pathway, and hepatocyte apoptosis. MET combined with exercise training significantly alleviated hepatic lipid accumulation by suppressing BiP expression, the central regulator of ER homeostasis, and its downstream PERK-eIF2*α*-ATF4 pathway, as well as upregulated the AMPK-Nrf2-HO-1 signaling pathway. Moreover, the combination of exercise and MET displayed protective effects on hepatocyte apoptosis by downregulating Bax expression and TUNEL-positive staining, restoring the balance of cleaved-caspase-3 and caspase-3, and improving the antioxidant defense system to prevent oxidative damage in db/db mice.

**Conclusion:**

Compared to MET or exercise intervention alone, the combined exercise and metformin exhibited significant effect on ameliorating hepatic steatosis, inhibiting oxidative and ER stress-induced hepatocyte apoptosis via improving the capacity of the antioxidant defense system and suppression of the PERK-eIF2*α*-ATF4 pathway. Furthermore, upregulation of AMPK-Nrf2-HO-1 signaling pathway might be a key crosstalk between MET and exercise, which may have additive effects on alleviating hepatic lipid accumulation.

## 1. Introduction

The liver is a critical organ in the systemic metabolism, including the regulation of glucolipid metabolism. Hepatic lipid metabolism abnormalities are a cause of nonalcoholic fatty liver disease (NAFLD). NAFLD and nonalcoholic steatohepatitis (NASH) are common in patients with T2DM [1], and approximately 70% of persons with T2DM have a fatty liver. T2DM and NAFLD coexist together to cause even more serious metabolic disease [2].

During the progression of T2DM, hepatic carbohydrate and lipid biosynthesis fluxes become elevated, which leads to cellular stress and hepatic injury thereby having hepatic lipid accumulation. As a key organ of lipid homeostasis, the liver is responsible for orchestrating cholesterol synthesis, lipid droplet formation, triglycerides export, and subsequent redistribution to other tissues [3]. Studies have indicated that in patients with T2D, liver lipogenesis is abnormally increased, and fatty acid oxidation and triglyceride secretion via very low-density lipoprotein (VLDL) are decreased [4–6]. The disruption of these processes may precipitate the retention of fat within the liver and the subsequent development of NAFLD. In addition, peripheral insulin resistance increases fatty acid release from adipose tissue [7], and the hepatic uptake of fatty acids also upregulated under the insulin resistance status [8]. Thus, T2D patients exhibit lipotoxicity and inflammation, and fibrosis in liver tissue is more due to the excessive hepatic FFA influx [9]. Excessive FFA oxidation in the liver further increases oxygen consumption and is the main resources of ROS production, thereby inducing oxidative stress [10] and endoplasmic reticulum stress (ERS) that result in hepatocellular damage and apoptosis. The PERK-eIF2*α*-ATF4 pathway is one of the major ER stress pathways and is required for cell survival. The ER stress sensor protein kinase R-like endoplasmic reticulum kinase (PERK) induces apoptosis under acute or prolonged ER stress conditions [11]. Under ER stress, PERK is dimerized through trans-autophosphorylation to activate eukaryotic translation initiation factor 2*α* (eukaryotic initiation factor 2*α*, eIF2*α*), temporarily blocking the translation initiation of proteins and alleviating the folding load of ER proteins. When the intracellular protein homeostasis cannot be restored, the expression of transcription factor CCAAT/enhancer binding protein homologous protein (CHOP) will be enhanced and further trigger apoptosis [12].

Metformin (MET) has been reported to decrease hepatic lipid levels and reduce glucose production in patients with T2DM [13, 14]. Despite the health benefit of MET, previous studies have also shown a few side effects of MET, such as lactic acidosis, renal impairment, and diarrhea [15–17]. Consequently, combination therapy, such as MET combined with other drugs or exercise intervention, has been proposed to eliminate the side effects of MET [18–20]. Exercise training can be effective intervention when treating NAFLD by decreasing intrahepatic fat content, enhancing mitochondrial function of fatty acids oxidation, attenuating hepatocyte apoptosis, and improving insulin sensitivity [21–24]. Although both MET and exercise interventions are known to phosphorylate AMPK to maintain glucose homeostasis, the cooperative effect of these two treatments it still debated. Recently, Ortega et al. reported that compared to exercise intervention alone, a combination of MET and exercise treatments accelerated a greater glucose disposal rate and increased a 43% (non-significant) in insulin sensitivity [25]. However, a study from Pilmark's group showed that a combination of treatments was not superior to exercise intervention alone in improving postprandial glucose [26]. Although MET or exercise showed the benefit in modifying hepatic fatty acid metabolism and was considered a therapeutic option for NAFLD [27–29], it is still lack of evidence on how MET or exercise interactively affects the hepatocellular lipid accumulation in NAFLD. Thus, the present study is aimed at determining the independent and combined effects of MET and exercise on hepatic lipid accumulation oxidative and ER stress, and hepatocyte apoptosis in diabetic mice.

## 2. Materials and Methods

### 2.1. Experimental Animals and Study Design

Forty male BKS-db/db mice were used in the experiments (age, 8 weeks; weight, 39 ± 12 g; Jiangsu Ji Cui Yao Kang Technology Co., Ltd, Nanjing, China). The experimental mice were housed in the Experimental Animal Center of Jiangsu Academy of Agricultural Sciences at 20 ± 2°C and 50% ± 5% humidity under a 12/12 h light/dark cycle with freely available food and water. All experiments and procedures were performed according to the National Institute of Health guidelines (NIH Publications No. 8023, revised in 1978) and were approved by the Animal Ethics and Welfare Committee of Nanjing Sport Institute (Approval No. 2019-010).

After 7 days of acclimation, animals that satisfied the diagnostic criteria for diabetes (random blood glucose ≥13.8 mmol/L (250 mg/dL)) [30] were randomly divided into the following four groups: control group (CON), exercise group (EX), metformin treatment group (MET), and exercise combined with the MET intervention group (EM). Mice in the MET and EM groups were orally gavaged daily with MET (Shanghai Squibb Co., Ltd) with the dosage of 300 mg/kg/day based on previous reports [31]. The other groups were given pure water intragastrically as a placebo.

### 2.2. Training Protocol

Mice in the EX and EM groups were trained at moderate intensity on a treadmill of 5 days/week for 8 weeks. Detailed training protocol including the running speed and duration is shown in Table 1 [32, 33]. The running speed accelerated slowly from the rest to the final speed within 5 min.

### 2.3. Sample Preparation

The mice's body weight was recorded once a week during the experimental period. After 8 weeks of intervention, all mice were anesthetized with 10% pentobarbital sodium (Sigma, USA) at a dose of 0.1 mL/10 g after fasting 12 h. Then, the blood sample was collected by eyeball extirpating and centrifuged at 4°C for 15 min at 3500 × *g*, and the supernatant was harvested and stored directly into –80°C for more analysis. The liver tissues were quickly excised, rinsed with cold physiological saline, and then fixed with 10% paraformaldehyde solution for histopathological assessment or stored at –20°C for further analysis.

### 2.4. Serum Biochemical Test

Commercial kits (Jian Cheng Bioengineering Institute, Nanjing, China) were used to test serum lipid of total cholesterol (TC) (A111-1-1), triglyceride (TG) (A110-1-1), low-density lipoprotein cholesterol (LDL-C) (A113-1-1), high-density lipoprotein cholesterol (HDL-C) (A112-1-1), free fatty acid (FFA) (A042-2-1), aspartate aminotransferase (AST)(C010-2-1), and alanine aminotransferase (ALT) (C009-2-1) according to the manufacturer's protocol.

### 2.5. Measurement of Liver Oxidative Stress

The liver tissue homogenate was obtained to detect total antioxidant capacity (T-AOC), malondialdehyde (MDA), catalase (CAT), and superoxide dismutase (SOD), by using the relative assay kits from Boxbio, Beijing, China.

### 2.6. Hematoxylin-Eosin and Oil Red O Staining

Hematoxylin and eosin (H&E) staining was conducted to observe the structural integrity of liver cells, hepatocyte arrangement, and nuclear and cytoplasmic status, reflecting the pathological morphology of the liver. Briefly, liver tissues were sliced into 4 *μ*m sections after fixing with 4% paraformaldehyde and embedding with paraffin. Then, tissues were deparaffinized and stained with H&E (Biosharp, BL702A) or oil red O (Sigma; MAK194). Images were captured with a microscope (Zeiss, Axio Imager A2). Five visual fields were randomly selected from each section to observe the pathological morphology of the liver.

### 2.7. Immunohistochemistry and Immunofluorescence

To evaluate the expression of superoxide dismutase-1 (SOD1) and the binding immunoglobulin protein (BiP), liver samples were embedded into paraffin and stored at room temperature. The liver samples were sliced at 5 *μ*m thickness and stained with SOD1 antibody (1 : 200, Cell Signaling, USA) and BiP antibody (1 : 200, Bioworld, USA). The tissues were observed and photographed under the microscope to count the area of positive SOD1 and BiP staining in the visual field. The terminal deoxynucleotide-transferase- (TdT-) mediated dUTP nick end labeling (TUNEL) method was used to histologically identify hepatocyte apoptosis, based on the manufacturer's instructions (Beyotime, Shanghai, China). The nuclei were stained with 4′,6-diamidino-2-phenylindole (DAPI, Beyotime, Shanghai, China) after the TUNEL assay. The apoptotic cells were visualized using a light microscope (Zeiss Axio Imager A2, Germany). Five visual fields were randomly selected from each slice to count the number of apoptotic cells. The percentage of TUNEL-positive cells/total number of cells ×100% was analyzed.

### 2.8. Western Blotting

Total proteins of liver tissues were extracted in radioimmunoassay precipitation lysis buffer containing 1% benzoyl fluoride (Beyotime Biotechnology, Shanghai, China). BCA protein detection kit (Epizyme, Shanghai, China) was used to quantify these proteins. Western blotting was carried out as described previously. Briefly, 30–50 *μ*g proteins were subjected to 10%–12% (w/v) SDS-PAGE gel. The separated proteins were electroblotted and transferred to a PVDF membrane (Millipore, Shanghai, China). The membrane was blocked with TBST containing 5% nonfat milk or bovine serum albumin, incubated with primary antibodies overnight at 4°C. After washing with TBST, the membrane was incubated with secondary antibodies at room temperature. The protein blots were visualized by using an ECL system and the Image Lab detection system (BioRad, Hercules, CA). The following primary antibodies were used: SOD1 (1 : 1000, Cell Signaling, USA), BiP (1 : 1000, Bioworld, USA), p-PERK (1 : 1000, Affinity, UK), p-eIF2*α* (1 : 1000, Affinity, UK), ATF4 (1 : 1000, Proteintech, USA), CHOP (1 : 1000, Cell Signaling, USA), cleaved-caspase-3 (1 : 1000, Cell Signaling, USA), Bax (1 : 1000, Cell Signaling, USA), BCL2 (1 : 1000, Abcam, USA), p-AMPK (1 : 1000, Cell Signaling, USA), AMPK (1 : 1000, Cell Signaling, USA), Nrf2 (1 : 1000, Proteintech, USA), HO-1 (1 : 1000, Abcam, USA), and GAPDH (1 : 20000, Proteintech, USA). The following secondary antibodies were used: goat anti-mouse IgG H&L (HRP) (1 : 10000, Proteintech, USA) and anti-rabbit IgG H&L (HRP) (1 : 3000, Cell Signaling, USA).

### 2.9. Statistical Analysis

Data are presented as the mean ± standard error of means (SEM) using GraphPad Prism 5.01 software (San Diego, CA, USA). Multiple group differences were evaluated by two-way analysis of variance (ANOVA), followed by Dunnett's multiple comparison test. *P* < 0.05 was considered as significant differences.

## 3. Results

### 3.1. General Effects of MET with or without Exercise in db/db Mice

**Figure 1:**
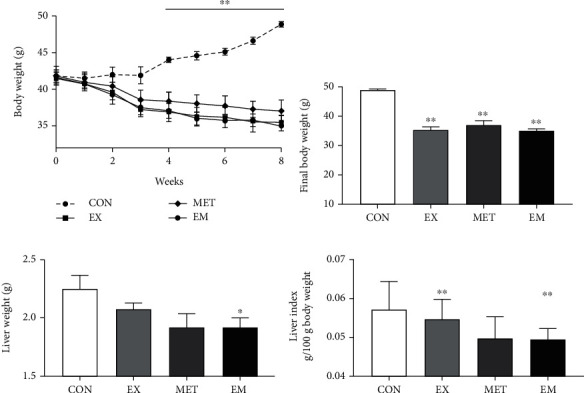
Mice's body weight and liver weight in each experimental group. (a) Mice's body weight throughout the experiment period (^∗∗^*P* < 0.01 vs. CON, line means from the 4-week to 8-week time). (b) Final body weight. (c) Liver weight. (d) Liver index. ^∗^*P* < 0.05 and ^∗∗^*P* < 0.01 vs. CON. All data are reported as the means ± SEM; *n* = 7 − 10.

Mice's body weight was monitored once a week and is presented in Figure 1. During the experimental period, compared with the normal control (NC) mice, the db/db mice were characterized by significantly higher body weight and liver weight (supplementary figure 1(a-1c)). The db/db mice in the CON group showed significant increase in their body weight (*P* < 0.01) from 4 weeks to 8 weeks compared with the baseline. By contrast, mice in the EX, MET, and EM groups significantly decreased their body weight (*P* < 0.01) from the 4-week exercise to the end of the experiment (Figure 1(a)). At the completion of the 8-week exercise or metformin intervention, the body weight (*P* < 0.01) was significantly lower in the EX, MET, and EM groups than that of in the CON group (Figure 1(b)). Moreover, the liver weight tended to decline after intervention in the EX, MET, and EM groups compared to the CON group, but only the EM group (*P* < 0.05) showed significant reduced (Figure 1(c)). There was no significant difference in the liver weight after adjusting for bodyweight (*P* > 0.05) among the groups (Figure 1(d)).

### 3.2. Combined Effect of MET and Exercise Ameliorated Dyslipidemia in db/db Mice

The db/db mice exhibited abnormal fasting blood glucose (supplementary figure 1(e, 1f)) and lipid levels. After 8 weeks of intervention, the serum TG and FFA levels (*P* < 0.05) in the EX, MET, and EM groups significantly decreased compared to the CON group (Table 2), whereas there was no significant difference in serum CHOL and HDL-C (*P* > 0.05) following metformin and/or exercise treatments. Notably, the levels of serum TG, LDL-C, and FFA (*P* < 0.05) in the EM group were significant reduced compared to the CON group. These findings suggested that the combined effect of exercise and MET has a more remarkable effect on alleviating dyslipidemia in db/db mice.

Data are means ± SEM. ^∗^*P* < 0.05 and ^∗∗^*P* < 0.01 vs. CON. 7-10 animals per group were used. CHOL: total cholesterol; TG: triglyceride; HDL-C: high-density lipoprotein-cholesterol; LDL-C: low-density lipoprotein cholesterol; FFA: free fatty acid.

### 3.3. Independent and Combined Effects of Exercise and MET Alleviated Lipid Deposition in db/db Mice

**Figure 2:**
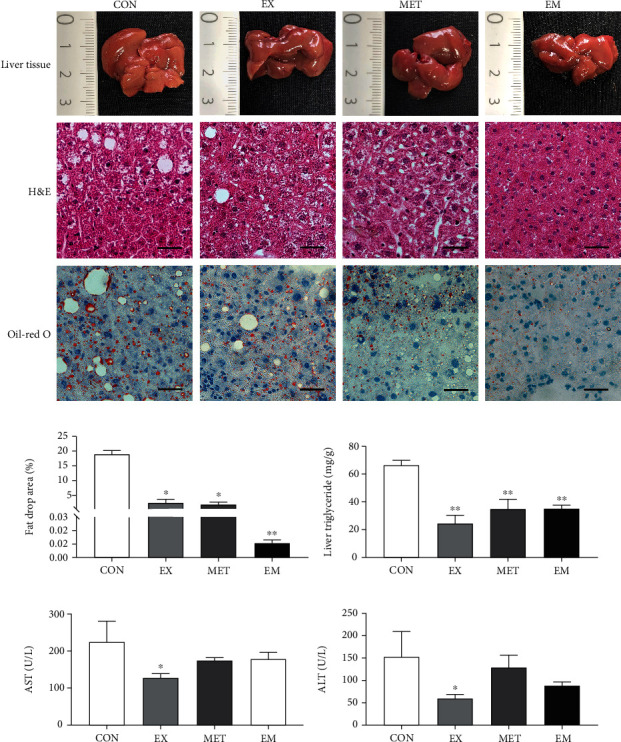
Effect of exercise and/or MET on liver lipid deposition and liver injury. (a) Liver tissue picture, H&E stained liver tissues, and oil red O staining showing lipid accumulation in the hepatic tissue of mice (scale bar: 50 *μ*m). (b) Fat drop area (%). (c) Hepatic levels of triglycerides (TGs).(d) AST level. (e) ALT level. ^∗^*P* < 0.05 and ^∗∗^*P* < 0.01 vs. CON. All data are expressed as mean ± SEM; *n* = 6 − 8.

H&E and oil red O staining of liver tissues were used to observe the histopathological changes and to visualize lipid droplets in the liver tissue, respectively (Figure 2(a)). The db/db mice exhibited hepatocyte ballooning and disordered arrangement. However, hepatocytes of the mice in the EX and EM groups exhibited a complete structure, orderly arrangement, and normal nuclear size after the 8-week exercise or exercise combined with metformin intervention. Moreover, although the db/db mice had similar level in liver glycogen content compared with normal control mice (supplementary figure 1(d)), in the CON group, a large number of lipid droplets were observed in the liver section. The percentage of lipid droplets' area in hepatocytes and hepatic TG content (*P* < 0.05) was significantly decreased in the EX, MET, and EM groups compared to the CON group (Figures 2(b) and 2(c)). Compared with db/db mice, a significant increase in liver glycogen content was only observed in the EX group. These findings demonstrated that independent and combined effects of exercise and MET were able to successfully alleviate lipid deposition in db/db mice.

Additionally, serum ASL and ALT parameters were measured in the four experimental groups (Figures 2(d) and 2(e)). After 8-week intervention, the serum AST and ALT were (*P* < 0.05) significantly reduced only in the EX group, whereas no significant difference (*P* > 0.05) in the MET or EM group by contrast to the CON group was observed. These results suggested that exercise exerts a greater effect on liver damage.

### 3.4. MET and Exercise Improved the Oxidative Stress in the Liver of db/db Mice

**Figure 3:**
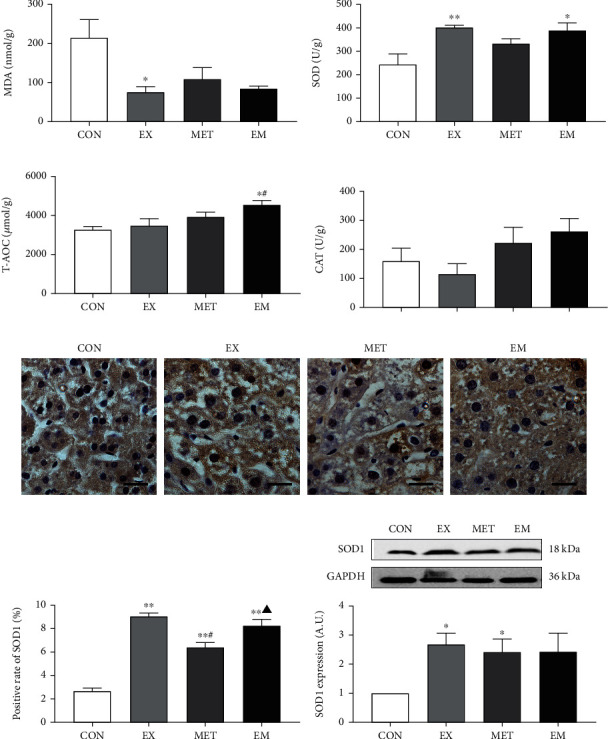
Effect of exercise and/or MET on makers of oxidative status in the liver tissue. (a) MDA enzyme activity level. (b) SOD enzyme activity level. (c) T-AOC enzyme activity level. (d) CAT enzyme activity level. (e) The superoxide dismutase-1 (SOD1) immunohistochemical staining of liver tissue (magnification ×400, scale bar: 20 *μ*m.). (f) Positive rate of SOD1 (%) in each group. (g) SOD1 expression level. ^∗^*P* < 0.05 and ^∗∗^*P* < 0.01 vs. CON, ^#^*P* < 0.05 vs. EX, ^▲^*P* < 0.05 vs. MET. All data are expressed as mean ± SEM; *n* = 8 − 10.

The effect of MET and/or exercise intervention on liver oxidative stress parameters is presented in Figure 3. The status of oxidative stress in the diabetic liver was evaluated by measuring the levels of oxidative stress factors (MDA) and antioxidant factors (SOD, T-AOC, and CAT). Compared to the CON group, the levels of MDA in liver tissue were significantly lower (*P* < 0.05) in the EX group (Figure 3(a)), and the levels of antioxidant factors SOD and T-AOC (*P* < 0.05) were significantly increased in the EM group (Figures 3(b) and 3(c)). However, there was no significant difference in CAT level (*P* > 0.05) following exercise and/or metformin treatments (Figure 3(d)). Furthermore, the result of SOD1 by ELISA detection was consistent with the immunohistochemistry and western blot (Figures 3(e)–3(g)). As shown in Figure 3(e), the relative fluorescence intensities of SOD1 (*P* < 0.01) in the EX, MET, and EM groups were significantly higher than in the CON group. Western blotting results demonstrated that the expression of SOD1 protein was significantly enhanced (*P* < 0.05) in EX and MET groups, compared with the CON group (Figure 3(g)). These results indicated that exercise, MET, and a combination therapy had equivalent effects in reducing the oxidative stress factors level and increasing antioxidant factors level in the diabetic liver.

### 3.5. Exercise Combined with MET Inhibited the PERK-eIF2*α*-ATF4 Signaling Pathway in db/db Mice

**Figure 4:**
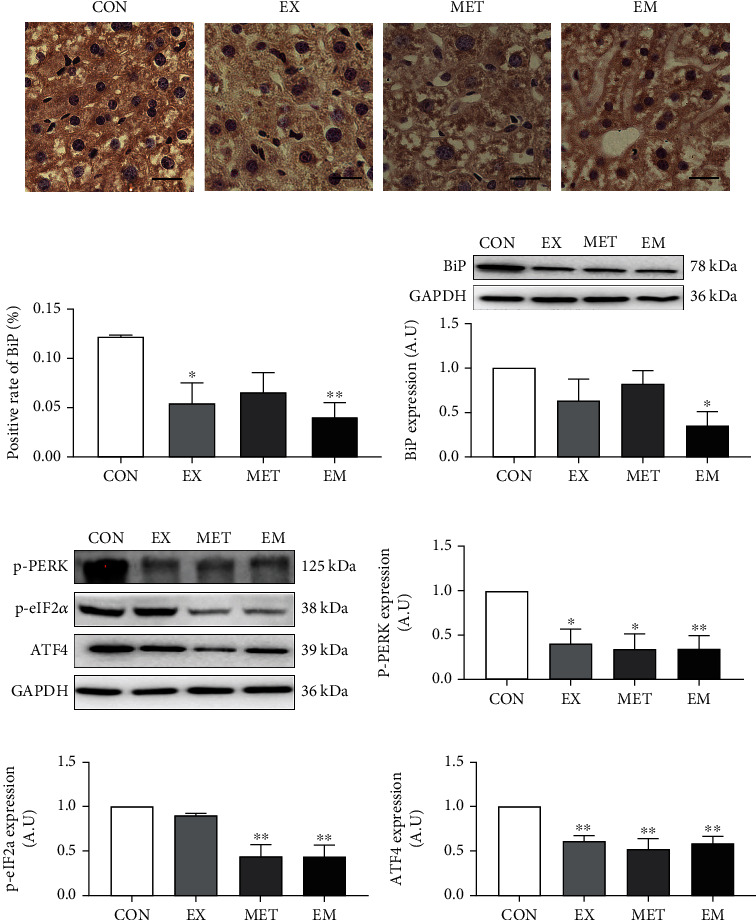
Effect of exercise and/or MET on hepatic endoplasmic reticulum (ER) stress. (a) The binding immunoglobulin protein (BiP) immunohistochemical staining of liver tissue (magnification ×400, scale bar: 20 *μ*m.). (b) Positive rate of BiP (%) in each group. (c) BiP expression in mice liver by western blot. (d) Representative western blot maps of ER stress protein. (e) p-PERK expression level. (f) p-eIF2*α* expression level. (g) ATF4 expression level. ^∗^*P* < 0.05 and ^∗∗^*P* < 0.01 vs. CON. All data are expressed as mean ± SEM; *n* = 6 − 8.

The immunohistochemical staining and western blotting for BiP in the liver tissue after exercise and/or MET intervention are shown in Figures 4(a)–4(c). Immunohistochemistry with anti-BiP antibody revealed strong BiP expression in db/db liver tissue. The staining intensity of the liver BiP was significantly reduced (*P* < 0.05) in the EX and EM groups compared to the CON group. The western blot assay also demonstrated that the expression of the BiP was significantly lower (*P* < 0.05) in the EM group than in the CON group (Figure 4(c)). Additionally, western blot analysis results (Figures 4(d)–4(g)) demonstrated that the expressions of p-PERK and p-eIF2*α* were all significantly downregulated (*P* < 0.05) in liver tissue in MET and EM groups compared with the CON group. The activated PERK promoted rapid phosphorylation of eIF2*α* and enhanced ATF4 expression, which is closely related to hepatocyte apoptosis. Western blot examination demonstrated that the ATF4 expression (*P* < 0.01) was markedly reduced with exercise, MET, and combined intervention. These results indicated that MET combined with exercise inhibited the PERK-eIF2*α*-ATF4 signaling pathway in db/db mice, and this effect was more effective than exercise or metformin intervention alone.

### 3.6. Combination of MET and Exercise Reduced Hepatocyte Apoptosis in db/db Mice

**Figure 5:**
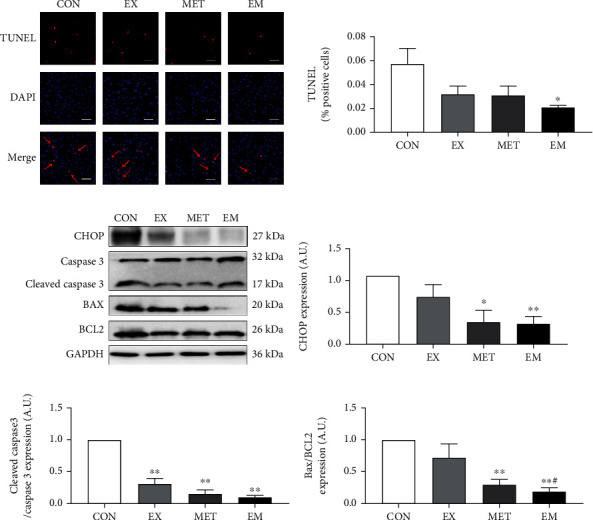
Effect of exercise and/or MET on hepatocyte apoptosis. (a) TUNEL, DAPI, Merge pictures (magnification ×400, scale bar: 20 *μ*m), and TUNEL-positive cell rate (%) (red arrow). (b) The western blot maps of proteins involved in the apoptotic process. (c) CHOP expression level. (d) Cleaved-caspase-3/caspase-3 expression level. (e) Bax/Bcl2 expression level. ^∗^*P* < 0.05 and ^∗∗^*P* < 0.01 vs. CON, ^#^*P* <0.05 vs. EX. All data are expressed as mean ± SEM; *n* = 7 − 10.

Immunofluorescence was performed to detect apoptosis in the liver tissue. TUNEL staining was clearly visible in the liver sections of the hepatic cells in the db/db mice (Figure 5(a)). However, the percentage of visualized apoptotic cells was significantly decreased (*P* < 0.05) in the EM group compared to the CON groups. The apoptosis protein expression exhibited that metformin and/or exercise intervention significantly inhibited ER-induced hepatocyte apoptosis by downregulating the ratio of cleaved-caspase-3 and caspase-3 protein (*P* < 0.01) level (Figure 5(d)). Consistent with this finding, western blotting demonstrated that the expression of CHOP and Bax/BCL2 protein levels (*P* < 0.05) was significantly reduced by MET treatment alone or MET combined with exercise intervention. Taken together, these data showed that the combination of MET and exercise was more effective at preventing the hepatocyte apoptosis in db/db mice than either intervention alone.

### 3.7. Combination of MET and Exercise Enhanced the Hepatic AMPK-Nrf2-HO-1 Pathway in db/db Mice

**Figure 6:**
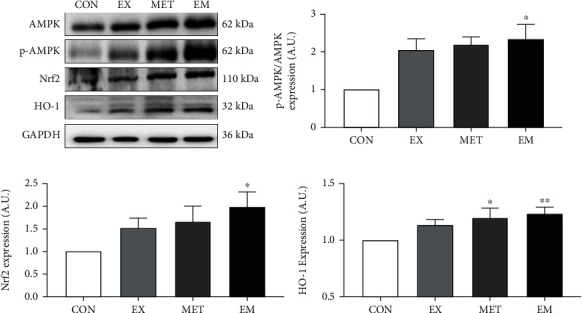
Effect of exercise and/or MET on hepatic AMPK-Nrf2-HO-1 pathway. (a) Protein maps of AMPK, p-AMPK, Nrf2, and HO-1 in the liver. (b) p-AMPK/AMPK expression level. (c) Nrf2 expression level. (d) HO-1 expression level. ^∗^*P* < 0.05 and ^∗∗^*P* < 0.01 vs. CON. All data are expressed as mean ± SEM; *n* = 7 − 10.

To further investigate the molecular mechanism underlying the MET and/or exercise-mediated inhibition of apoptosis, the liver protein expression of p-AMPK/AMPK ratio, Nrf2, and HO-1 is shown in Figure 6. Western blot analysis indicated that MET combined with exercise significantly increased the protein expression of p-AMPK/AMPK ratio (*P* < 0.05) and its downstream antioxidant gene Nrf2 and HO-1 expression (*P* < 0.05) compared with the CON group, while the AMPK-Nrf2-HO-1 pathway protein expression was not significantly identified (*P* > 0.05) with exercise intervention alone compared with the CON group. These data indicate the favorable effect of MET and exercise combination on upregulating the Nrf2 protein accumulation, leading to an enhancement of the antioxidant defense system to prevent oxidative damage in db/db mice.

## 4. Discussion

This study explored the independent and combined effects of MET with exercise training in the suppression of NAFLD and its underlying mechanisms in the context of diabetes. We demonstrate that both exercise and MET have beneficial effects for treating hepatic steatosis in diabetic mice, and that the combined exercise and MET showed greater benefit, mainly through reducing oxidative and ER stress (PERK-eIF2*α*-ATF4 pathway) and mediated hepatic apoptosis and enhancing the antioxidant defense system via AMPK-Nrf2-HO-1 pathway.

Exercise and MET are both found to have benefits on weight loss and health promotion [34, 35]. In the current study, MET significantly downregulated the increased body weight gain in db/db mice; although, the mechanisms by which MET suppresses body weight gain remain to be elucidated. Previous studies have reported that MET induces weight loss by improving insulin sensitivity, regulating fat oxidation, and affecting gut flora [36, 37]. Recent studies in both animal and human models have demonstrated that MET is an effective weight-reducing medication by elevating GDF-15 levels, which works as a “weight watcher” to maintain homeostasis [38, 39]. Additionally, a significant decrease in body weight was observed when exercise training was performed alone or in combination with MET treatment. This indicated that both exercise and MET can effectively inhibit the increased changes in body weight in db/db mice.

In a T2D liver, not only higher levels of glycogenolysis and gluconeogenesis are detected but also increased cholesterol and triglyceride synthesis [40]. Consistent with this characteristic, we observed a high level of hepatic TG associated with increased serum TG and LDL levels in db/db mice. Reducing lipotoxicity is considered important for preventing and/or reversing T2D. In the treatment of T2D, it is known that MET inhibits hepatic glucose production by suppressing gluconeogenesis [41] and increasing skeletal muscle glucose uptake. Inhibition of hepatocyte lipid synthesis by MET may contribute to phosphorylate 5′ AMP-activated protein kinase (AMPK) in liver tissue. Activated AMPK will suppress the expression of aminocylopropane-1-carboxylate (ACC), fatty acid synthase (FAS), and sterol regulatory element-binding protein 1 (SREBP-1) to shift liver lipid metabolism away from FFA synthesis and increase the level of lipolysis. MET can also increase fatty acid oxidation through activation of carnitine palmitoyltransferase I (CPT-1) [41, 42]. The underlying mechanism involves upregulated *β*-oxidation and lipogenesis, which are also associated with activating the AMPK signaling pathway [21]. Consistent with these studies, the current study demonstrated that liver lipid deposition in db/db mice was significantly alleviated by 8-week MET treatment with exercise intervention, as evidenced by decreased serum and hepatic TG levels, as well as a lower fat droplet area measured by oil red stain in the EM group. Although the effects of MET plus exercise on the liver is still largely unknown [43], we showed that intrahepatic fat accumulation was lowered after combined MET and exercise compared to MET or EX alone and suggest that exercise or MET alone can alleviate lipid deposition in diabetic mice, while the combined effect of exercise and MET seems to confer greater benefit.

Excessive accumulation of lipid droplets in nonadipose tissues such as liver or skeletal muscle can lead to cell dysfunction and death, named lipotoxicity [44]. This process can be accompanied by abnormal ROS production, membrane damage, lipid peroxidation, and decrease in intracellular antioxidants [21]. Next, we examined the effects of MET and exercise on oxidative stress level in diabetic livers. The present results addressed the level of antioxidant enzymes and gene expression such as superoxide dismutase (SOD), (T-AOC), and catalase (CAT) that were significantly upregulated in db/db mice after MET combined with exercise intervention, while malondialdehyde (MDA) content was significantly declined. SOD is an enzyme that facilitates the conversion of anionic superoxide (*o*_2_^−^) to hydrogen peroxide (H_2_O_2_) and O_2_ [45]. Our data showed that the fluorescence intensities and protein expression level of SOD1 were restored in the liver after MET combined with exercise treatment. These results demonstrated that administration of metformin combined with exercise has significant preventive and therapeutic effects on liver fat deposition by reducing the oxidative stress level and improving antioxidants status.

Multiple researchers showed that oxidative stress could trigger ER stress and mitochondrial apoptotic pathways. The ER chaperone protein BiP is a central regulator of ER homeostasis. During ER stress, the ER transmembrane sensors PERK, IRE-1*α*, and ATF6 were dissociate from BiP and subsequently activated. In this study, the BiP expression in the diabetic liver was significantly attenuated by MET and exercise treatment. Prolonged high level of ER stress will lead to cellular damage via the apoptotic signaling pathways and then initiate the unfolded protein response (UPR). The PERK-eIF2*α*-ATF4 axis is one of the major arms for mammalian UPR response activation and has been found to be involved in lipogenesis and steatosis regulation [46, 47]. If UPR response fails to alleviate the ER stress, the activated PERK-eIF2*α*-ATF4 pathway will promote apoptosis, marked by the increased expression of CHOP. Cao et al. demonstrated the association between the PERK-ATF4-CHOP pathway and hepatocyte apoptosis in the accumulation of saturated fatty acids in human liver cells [48]. Moreover, Koo and Han recently reported that alleviating ER stress by regulating downstream signaling molecules can be an effective treatment of NAFLD, which suggested that there is a strong correlation between ER stress and liver disease progression [49]. Consistent with these studies, this study found that abnormal lipid accumulation in db/db mice is closely related to a higher level of liver ER stress, and that the independent and combined effects of exercise and MET significantly reduce excessive lipotoxicity by inhibiting ER stress through the PERK-eIF2*α*-ATF4 pathway to reverse diabetic associated damage.

We next examined the hepatocyte apoptosis levels in db/db mice in response to MET and exercise interventions. In the mitochondrial-mediated apoptosis, excessive FFA content and oxidative stress increase Bax synthesis and induce the mitochondrial PT pore open and cytochrome C release. Thus, the occurrence of apoptosis is directly related to the ratio of the Bcl-2/Bax protein complex [50, 51]. We found that combined MET and exercise treatment significantly inhibited the higher level of TUNEL-positive staining and the ratio of the Bcl-2/Bax protein complex in db/db mice. Moreover, the mitochondrial apoptotic pathway would result in the activation of caspases signaling cascade [52, 53]. We found that MET and exercise intervention significantly attenuated ER-induced hepatocyte apoptosis by restoring the balance of cleaved-caspase-3 and caspase-3. Cleaved-caspase-3 is the main cleavage enzyme that promotes apoptosis [54]. The protective effects of MET and/or exercise on hepatocyte apoptosis could be partly attributed to the activation role of AMPK and related antiapoptotic signaling pathways. Recently, Zou et al. reported that the underlying mechanisms of alleviating the pathological changes in livers induced by high-fat diets through exercise may be related to SIRT1/AMPK signaling pathways [55]. Zhao et al. also showed the important role of an AMPK-caspase-6 axis in regulating liver damage in NASH [56]. We found that MET and exercise significantly enhanced the AMPK phosphorylation level and the expression of Nrf2-HO-1 signaling pathway to inhibit liver cell apoptosis and reduce liver damage, indicating the involvement of activated AMPK in inhibiting the hepatocyte apoptosis by MET and exercise intervention. In addition to the mitochondrial apoptotic pathway, mitochondria-associated ER membrane (MAM) integrity has been shown to be closely related to lipid metabolism, oxidative and ER stress, and apoptosis in diabetic mice [57–59]. The beneficial effects of MET treatment plus exercise on hepatocyte apoptosis are likely to improve mitochondrial function and MAM quantity. However, the specific mechanisms of these processes in the prevention and treatment of NAFLD need to be further clarified.

## 5. Conclusions

**Figure 7:**
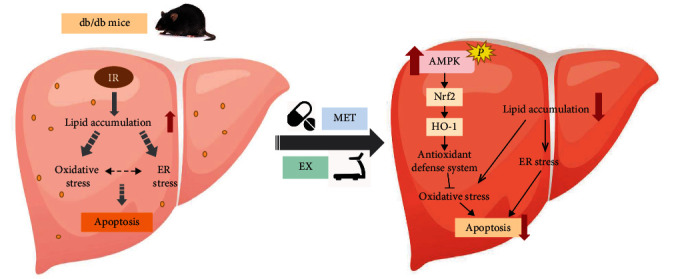
The potential mechanism underlying the additive effects of MET and exercise on alleviating lipotoxicity induced hepatocyte apoptosis.

In conclusion, this study demonstrated that lipid deposition in the liver of diabetic mice is associated with oxidative damage and activation of the PERK-eIF2*α*-ATF4 signaling pathway. The combination of MET and exercise exhibited additive effects on ameliorating hepatic steatosis, inhibiting oxidative and ER stress-induced hepatocyte apoptosis via improving the capacity of the antioxidant defense system. Considering metformin and exercise intervention which independently increased AMPK, the upregulation of AMPK-Nrf2-HO-1 signaling pathway might be the key mechanism of metformin and exercise synergistic effect (Figure 7).

## Figures and Tables

**Table 1:** Training protocol.

Week	0	1	2	3	4	5	6	7	8
Speed (m/min)	8	8	10	10	10	10	12	12	12
Time (min)	15	30	30	30	40	40	40	40	40

**Table 2:** Blood lipids of mice in the experimental groups ($\overset{-}{x}\pm s$).

Group	CON	EX	MET	EM
CHOL (mmol/L)	3.6517 ± 0.1442	3.2190 ± 0.1489	3.4320 ± 0.1185	3.1480 ± 0.0761
TG (mmol/L)	2.3760 ± 0.0919	1.5470 ± 0.0810^∗^	1.3360 ± 0.0771^∗∗^	1.4020 ± 0.0762^∗∗^
HDL-C (mmol/L)	2.3883 ± 0.0876	2.0817 ± 0.1174	2.1680 ± 0.0671	2.0433 ± 0.0668
LDL-C (mmol/L)	0.2550 ± 0.0197	0.2020 ± 0.0071	0.2050 ± 0.0169	0.1075 ± 0.0180^∗^
FFA (*μ*mol/L)	2472.8333 ± 45.6256	1143.6000 ± 84.9004^∗∗^	1432.7500 ± 79.3666^∗^	1343.4167 ± 49.8446^∗∗^

## Data Availability

The experimental data used to support the findings of this study are included within the article.

## Additional Points

*Declarations.* It is to be used for all articles, including articles with biological applications.

## Supplementary Materials

Supplementary Materials Supplementary Figure 1: general effects of MET with or without exercise in db/db mice compared with normal control mice (NC).
