# Supplementary material for: Exercise and Metformin Intervention Prevents Lipotoxicity-Induced Hepatocyte Apoptosis by Alleviating Oxidative and ER Stress and Activating the AMPK/Nrf2/HO-1 Signaling Pathway in db/db Mice
Source: Oxid Med Cell Longev. 2022 Sep 9;2022:2297268. doi: 10.1155/2022/2297268 (PMC9481363; doi:10.1155/2022/2297268)
Supplement: Supplementary Materials — Supplementary Figure 1: general effects of MET with or without exercise in db/db mice compared with normal control mice (NC). [file 2297268.f1.docx]

**
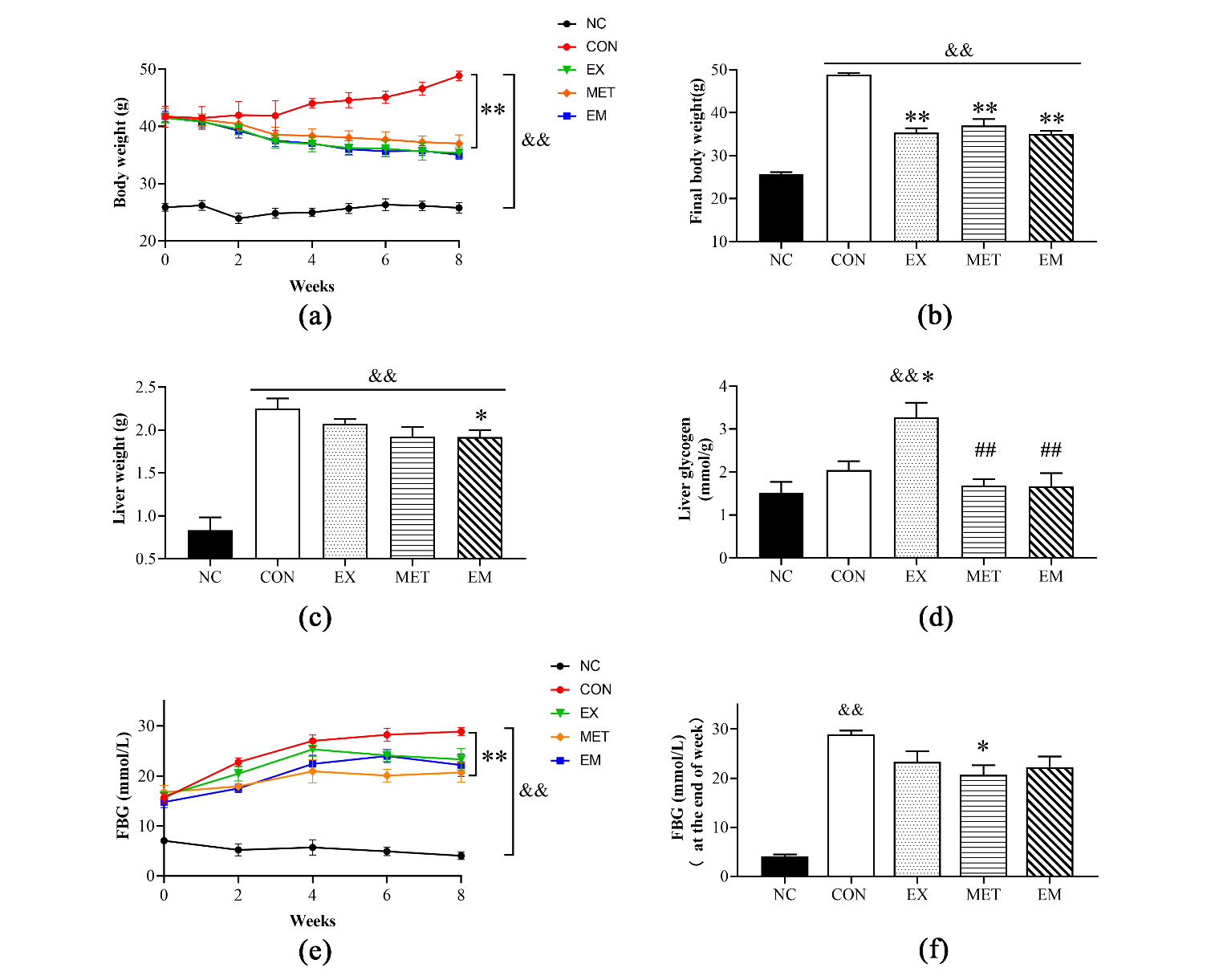
**Supplementary Figure 1

FIGURE 1.General effects of MET with or without exercise in db/db mice compared with normal control mice (NC). (a) Changes in body weight during the experiment; (b) Final body weight; (c) Liver weight; (d) Liver glycogen content; (e) Changes in fasting blood glucose (FBG) during the experiment; (f) Final fasting blood glucose. ^&&^*P* < 0.01 vs NC, ^*^*P* < 0.05, ^**^*P* < 0.01 vs CON, ^##^*P* < 0.01vs EX. All data are reported as the means ± SEM; 7-10 animals per group were used.
